# The induction process of new professionals in the Swedish ambulance service – a document analysis

**DOI:** 10.1186/s12909-026-09712-8

**Published:** 2026-06-16

**Authors:** Anna Hörberg, Kim Wallin, Helena Sjölin

**Affiliations:** 1 School of Health and Welfare, Dalarna University, Falun, SE 731 95 Sweden; 2 Department of Research, Development, and Education Region Kronoberg, Växjö, Kronoberg SE-35188 Sweden; 3 Faculty of Health and Life Sciences, Linnaeus University, Växjö, SE 351 95 Sweden; 4 School of Health Sciences, Faculty of Medicine & Health, Örebro University, Örebro, SE 70182 Sweden; 5 Ambulance Medical Service in Stockholm [Ambulanssjukvården i Storstockholm AB], Stockholm, Sweden

**Keywords:** Ambulance personnel, Document analysis, Education, Emergency medical service, Induction, New, Training

## Abstract

**Background:**

Working in the ambulance service is highly demanding, requiring new professionals to manage diverse, unpredictable patient situations from day one. New professionals often report limited self-confidence, emotional strain, and isolation, which can contribute to burnout, and turnover. Despite these challenges, in Sweden, education required for working in the ambulance service covers only a fraction of the essential competencies. Structured induction programmes are therefore critical for supporting new professionals. Although there is extensive knowledge about induction in hospital settings, little is known about induction in the ambulance service. Therefore, the aim of this study was to map the induction process of novice professionals in Swedish ambulance services.

**Methods:**

A descriptive study was conducted based on qualitative data from induction programme materials from 23 ambulance organisations, representing all 21 healthcare regions in Sweden. Data was analysed using document analysis.

**Results:**

Analysis yielded six categories describing the content of the introductory training; *Transportation and navigation; Cooperation and communication; Systematic work approaches and structures, Prerequisites for care and nursing; Safe healthcare environment; Organizational knowledge*, and a seventh category describing *Pedagogical approaches*.

**Conclusion:**

The findings of this study highlight a need for more evidence-based and pedagogically coherent induction programmes within ambulance services. Induction should support not only technical and clinical competence, but also interpersonal, ethical, and person-centred aspects of care, while fostering professional socialisation, belonging, and psychological safety. As this study was based on documentary analysis, further research is needed to examine how different induction designs influence learning, clinical competence, and patient safety in ambulance care.

## Background

Working in the ambulance service is recognized as having to manage a broad spectrum of patient care needs, often in unpredictable and rapidly changing environments from the very first day of employment [1–5]. Being new to this part of the healthcare system can be a challenging time and research on how novice nurses experience their first year emphasize feelings of loneliness, conflicting and sometimes unrealistic expectations of the own ability [6, 7]. Furthermore, it has been shown that new professionals in the ambulance service experience an imbalance between feeling prepared and not being prepared [8]. This imbalance may result in fatigue syndrome, burnout, and even post-traumatic stress syndrome (PTSD) [9, 10] as well as professionals leaving the ambulance service [11, 12]. To manage work in the ambulance service, staff need to possess emotional resilience [10] and receive extensive and specific educational preparations [3, 13, 14]. However, when evaluating how well nursing education prepares novice nurses for work in ambulance service, research shows that only eight out of 60 essential competencies are adequately covered in their training [3].

These circumstances underscore the importance of a structured and well-designed introductory period for novices entering the ambulance service. The introductory period, i.e., initial period when an individual joins a new profession or new workplace and receives formal organisational support and competency assessment may be described using different terminology internationally, in this study it will hereafter be refer to as induction. Well -designed induction programmes provide novice professionals with the necessary support to navigate the complexity of emergency care such as the ambulance service [15]. Furthermore, structured induction has been reported to strengthen professional confidence, which is of essence specifically in situations where collegial support and resources are limited, such as in the ambulance service [16].

Existing research has predominantly examined how universities design and deliver educational programmes in ambulance care [5, 14], as well as students’ experience of their clinical placements [17], and the experiences of being new to the ambulance service [18]. However, there remains a notable lack of knowledge regarding how ambulance services themselves organize and enact their induction programmes for novice professionals.

In the literature, a recurring issue is the discrepancy between the knowledge and competences students acquire during their university studies and the knowledge and competencies required in real-world clinical practice. This discrepancy is commonly described as the *theory–practice gap* [19]. Although the theory–practice gap has been extensively discussed, existing research has not yet established how this gap can be effectively bridged. Instead, findings consistently indicate that while theoretical knowledge is essential, its practical application in complex and unpredictable clinical environments often receives insufficient attention [20, 21]. When students articulate the theory–practice gap, they frequently describe their initial encounters with clinical work as a *reality shock*—a sudden recognition that, despite feeling prepared during their education, they do not perceive themselves as adequately equipped for the demands of for example emergency care once they enter the profession [22]. Reality shock has been associated with negative outcomes, including increased turnover intentions and work-related ill health [11, 12].

A structured induction is essential for fostering a continuous learning process that enhances professional competence and security which may reduce reality shock and bridge a potential theory practice gap, enhance patient safety, and in the long run increase retention and a sustainable work-life [23–25]. Through a systematic mapping of the content of induction programmes, this study identifies the theoretical content and learning strategies used in introducing new professionals to the clinical reality of ambulance care. The results of this mapping can also reveal strengths and potential weaknesses that can be used to create research-based structured inductions for new professionals. To increase the opportunity for universities and ambulance services worldwide to approach each other and reduce the “theory-practice gap”, the aim of this study is to map the induction process of novice professionals in Swedish ambulance services.

## Method

### Study design

The study was conducted with descriptive design using qualitative data. Documents describing information and content of the induction programme for novice ambulance professionals in Sweden, furthermore, referred to as “induction document”, were analysed using document analysis inspired by Bowen [26]. The method involves a systematic evaluation of document content to gain understanding and develop empirical knowledge”.

### Setting and participants

Sweden is divided into 21 regions, and in most cases, ambulance services are operated under regional management. In addition to regionally managed services, four private providers have been contracted to deliver ambulance services in certain regions. In total, ambulance services in Sweden are provided by 25 different organizations. In line with Swedish legislations, emergency ambulances in Sweden are staffed with at least one registered nurse (RN) holding a bachelor’s degree in nursing. However, some Swedish healthcare regions demand at least one of the ambulances professionals to be a specialized RN holding an additional one-year master’s degree in nursing. Typically, an RN or specialist RN, works either with another RN or an emergency medical technician (EMT). In Sweden an EMT is a nurse assistant, whose formal competence includes basic life support and additional ambulance-specific training [27].

A total of 33 eligible different heads of operations were identified through the region’s websites and through personal contacts. These individuals were informed via email about the study design, and those who approved the implementation of the study within their unit were asked to provide contact details for the person responsible for the induction programme and sign a consent form. In addition to the initial email, two reminder emails were sent.

Twenty-three heads of operations, representing the 21 different regions, responded, signed the consent form, and provided contact details of the person responsible for the programme. These individuals were subsequently contacted via email and received information about the study, a description of the requested materials, and the signed consent form from their head of operations. Induction documents were obtained from (*n* = 23) ambulance services. The study has been reviewed by the Ethical Advisory Board in Southeast Sweden (Dnr 935–2023).

### Data collection

The documents were delivered digitally and organized into folders, one for each ambulance service. The number of documents per ambulance service varied and consisted of various types of informational materials, such as welcome letters, training and course plans, schedules for the introductory period, descriptions of training content, assessment forms, and checklists. Additional materials included emails, links to preparatory materials, intranet screenshots, and PowerPoint presentations from introductory lectures. One region requested to be interviewed about their introductory training programme due to insufficient documentation regarding their induction. The interview consisted of one question “tell us about the induction programme at the ambulance service?” and follow-up questions like “tell us more about that?” or “please elaborate”. No interview guide was used. The interview was conducted via Zoom, recorded, and transcribed verbatim. The transcribed material was treated as a document and analysed according to the document analysis according to Bowen [26]. All documents were stored on a password-protected and secure university server, accessible only to the researchers involved in the study.

### Data analysis

According to Bowen [26] the analysis begins by first skimming all the text, then reading it more carefully and finally interpreting the content in relation to the purpose of the study. Each author (AH, KW, HS) skimmed and then read through the entire material to gain an understanding and create a picture of the data. After a joint discussion, it was identified that the material could be divided into two parts, one with content from the introductory courses and one with descriptions of different pedagogical approaches. Codes were then identified from the text that described content and pedagogical approaches and sorted based on affinity.

For this sorting process a web-based Excel document was created, and all authors met in person to perform an initial sorting of the codes from three ambulance service induction documents. During this process, joint discussions were held regarding how the codes should be organised into subcategories labelled with descriptive terms. After establishing a shared analytical structure, the remaining documents were distributed among the three authors, who then analysed them individually using the same analytical approach applied to the initial three induction documents. All authors could see each other’s sorting during the work and communicated digitally when necessary. To ensure consensus, the research group also met physically on six occasions for discussions regarding sorting of codes, creation of subcategories and finally the merging of subcategories into categories. The final phase of the analysis resulted in eighteen subcategories and six categories, derived from the data that dealt with the content (Table 1). The descriptions of the different pedagogical approaches formed their own independent category, with ten subcategories describing different pedagogical approaches.

**Table 1:** Example of the analysis process

Codes	Subcategory	Category
Personal protection equipment	Personal safety measures	Safe healthcare environment
Traffic education	Driver training and vehicle knowledge	Transportation and navigation
RAKEL*	Information transfer	Cooperation and communication
PHTLS (prehospital trauma life support)	Assessment and triage	Systematic work approaches and structures
Personal values	Professional approach	Prerequisites for care and nursing
Wash routines for uniform	Local workplace information	Organizational knowledge
CPR-council webb-education	Webb-based or recorded material	Pedagogical approach

*Swedish national digital communications system used by the emergency services

## Results

The analysis of the content of the induction documents covering all 21 healthcare regions in Sweden resulted in six categories, Transportation and navigation, Cooperation and communication, Systematic work approaches and structures, Prerequisites for care and nursing, Safe healthcare environment and Organizational knowledge (Table 2). The analysis of the different pedagogical approaches resulted in the seventh category Pedagogical approaches (Table 3). 

**Table 2:** Data in categories and subcategories including number of organizations* with this specific content described in their induction documents

Categories	Transportation and navigation		Operation and communication		Systematic work approaches and structures		Prerequisites for care and nursing		Safe healthcare environment		Organizational knowledge	
	*n* (%)		*n* (%)		*n* (%)		*n* (%)		*n* (%)		*n* (%)	
Subcategories and frequencies	Driver training and vehicle knowledge	23* (100)	Information transfer	23* **(100)**	Prehospital incident management	21 (91)	Equipment	11 (48)	Dangerous substances	18 (78)	Central operational information	20 (87)
	Navigation systems and geographical knowledge	19 (83)	Healthcare and emergency service stakeholders	15 (65)	Assessment and triage	17 (74)	Medical devices	21 (91)	Healthcare hygiene	16 (70)	Local workplace information	15 (65)
					Processes and routines in healthcare	23* **(100)**	Medications	11 (48)	Personal safety measures	18 (78)	Digital workplace systems	17 (74)
							Professional approach	5 (22)	Ergonomics and transfer techniques	23* **(100)**		

*Total of organisations is *n* = 23, text presented in bold denotes content identified in all organisations’ induction programmes.

**Table 3:** Pedagogical approaches and number of organizations with this specific approach described in their induction documents

Assessment by tests *n* (%)	Authorization by a physician *n* (%)	Classroom lectures *n* (%)	Control by checklists *n* (%)	Mentor *n* (%)	Online material *n* (%)	Physical on-site visits *n* (%)	Practical skills training *n* (%)	Self-studies *n* (%)	Simulation exercises *n* (%)
11 (48)	12 (52)	16 (70)	18 (78)	8 (35)	17 (74)	12 (52)	18 (78)	6 (26)	13 (57)

In summary the induction documents varied in detail concerning both timespan and content of the induction. Some documents presented clear and well-defined timeframes for the induction and its components, ranging from four days to five weeks while others were less specific. The induction programmes all constituted theoretical education followed by practical training and “ride-along”, meaning that the new professional accompanied the ambulance professionals in their clinical work. The “ride-along” period ranged from two days to three weeks. In some documents, additional education occurring after six months, one year, or two years, were described. Furthermore, differences in induction content exist between professional categories; EMT’s were expected to be “ready to drive” after completing their induction programme, but this requirement was not as explicitly documented for RN’s. Additionally, some induction documents mentioned that individual adaptations were made based on the prior knowledge level of the novice professional.

The content of the categories will be further described in detail by each subcategory.

### Transportation and navigation

This category portrays the content of the induction that focuses on the technical, practical, and psychological perspectives of driving, and proper maintenance of the ambulance vehicle as well as mastering the navigation system and geographical knowledge. Transportation and navigation were one aspect of the induction that was covered by all (*n* = 23) ambulance services.

#### Driver training and vehicle knowledge

All ambulance services (*n* = 23) include some form of driver training, though variation in timing, content, and extent exists. Driver training ranged from multi-step programmes spanning months to single-day courses before employment. The basis in training objectives was vehicle familiarization, maintenance, and knowledge of equipment associated with safe driving, e.g., inspection of tire pressure and tread depth. The overall learning objective varied between independently driving a priority or supporting an experienced driver as an assistant. Some documents highlighted that the driver training was tailored in accordance with prior experience and competency levels.

Theoretical and practical components were integrated, with assessments before, during, and after training. Theoretical instruction covered traffic regulations and psychology, emphasizing safe driving behaviour and attitudes. Practical training included manoeuvring, highway-, winter-, night-, and urban driving, with increasing difficulty levels. Some organizations require heavy vehicle license post-training.

#### Navigation systems and geographical knowledge

Map reading and geographical knowledge were standard components in all (*n* = 23) organization’s documents, involving both digital and analogy navigation aids, and physical site visits. All ambulance services (*n* = 23) employed checklists for equipment and daily safety checks.

### Cooperation and communication

This category concerns training focusing on structures for communication and cooperation in the ambulance service. The content in the introductory documents addressed training in systematic and structured ways for transferring information using technical and analogue tools. The documents also described the focus on familiarization with, and understanding of, healthcare and emergency service stakeholders and their role and responsibility in relation to the ambulance service.

#### Information transfer

Almost all (*n* = 21) of the ambulance services report training in using various technical tools and systems for documenting patient care. Furthermore, different ways for information transfer and communication either with another ambulance or with collaborating organizations, i.e., fire department och police, using communication tools such as RAKEL (Radio Communication for Effective Command, a multiauthority radio communication used by ambulance and dispatch), or Paratus (a system used, via an in-vehicle computer, to manage and respond to incidents from a dispatch centre). Additionally, the use of structured protocols and algorithms for reporting patient status such as SBAR (Situation, Background, Assessment and Recommendation) were trained according to the documents.

#### Healthcare and emergency service stakeholders

A majority (*n* = 14) of ambulance services display the use of study visits and/or the provision of information regarding healthcare and emergency service stakeholders, including the police, fire and rescue services, emergency medical dispatch centre, maritime rescue, and aeromedical rescue were mentioned. Study visits also involved different hospital departments or units, physician support services, and municipal healthcare facilities. In some organizations, the purpose of these study visits was explicitly described as fostering an understanding of the different roles and responsibilities between the healthcare and emergency service stakeholders and the ambulance service. Regarding emergency departments, some descriptions emphasize a focus to facilitate participation in patient care within an emergency room.

### Systematic work approaches and structures

The content of the induction documents also contained information about training different systematic work approaches and structures used to identify care needs of the patient and to optimize prerequisites for care in the prehospital setting. The systematic work approaches involved prehospital incident management, structures to assure correct assessment and triage, and learning about specific processes, routines and medical guidelines.

#### Prehospital incident management

Nineteen services explicitly mention prehospital incident management training. The timing of this training varied from day 4 of the induction to 12 months post-employment. Where prehospital incident management training was not explicitly stated, related training in mass casualty incident management, interagency coordination, and structured ways for radio communication was described. For example, METHANE, (a structured way to communicate scene information: Major incident, Exact location, Type of incident, Hazards, Access, Number of casualties, Emergency services present and required).

#### Assessment and triage

Most services (*n* = 17) induction programmes explicitly mention some sort of training in prehospital assessment, triage, and different structured ways to assess patient needs. The structured ways described in the documents are presented and explained in Table 4.

**Table 4:** Frequency and explanation of the different structured ways to assess patient needs trained according to the induction material

Structure	Content	Frequency *n*/*N* (%)
AMLS	Emphasizes the use of the AMLS Assessment Pathway, a systematic tool for assessing and managing common medical conditions with urgent accuracy. Stands for: Advanced Medical Life Support	10/23 (43)
AOSP	A course developed as part of the Suicide Prevention in Stockholm County (SPIS) – a collaborative project between Police, Stockholm Fire Brigade, Ambulance care in Greater Stockholm Ltd, SOS-Alarm, The National Association for Suicide Prevention and Support for Families of Suicide Victims (SPES) and NASP. Stands for: Acute management Of Suicidal Person	3/23 (13)
APP	A Swedish model for the initial assessment of psychiatric patients in acute or prehospital settings. Stands for: Ambulance Psychiatric Priority	1/23 (4)
ATLS	A standardized, global course focused on the initial assessment and management of trauma patients. Stands for: Advanced Trauma Life Support	1/23 (4)
PEPP	A comprehensive course featuring case-based lectures, live-action video, hands-on skills stations, and small group scenarios. From infant modifications to critical diagnoses. Stands for: Pediatric Education for Prehospital Professionals	2/23 (9)
PHTLS	A continuing education programme for prehospital emergency trauma care. Stands for: Pre Hospital Trauma Life Support	8/23 (35)
RETTS	A decision support system for emergency care. Stands for: Rapid Emergency Triage and Treatment System	12/23 (52)
SAMPLE	A structure to obtain anamnesis and medical history from a patient. Stands for: Signs and symptoms, Allergies, Medications, Past medical history, Last oral intake and Events leading to	2/23 (9)
TERMA	A Swedish protocol for managing aggressive or violent patients in prehospital or emergency settings focusing on safety, communication, and de-escalation techniques. Stands for: Team, Evaluation, Response, Management, and Action.	1/23 (4)
(X)ABCDE	A term used in trauma care. It represents a prioritized approach to assessing and treating trauma patients. Stands for: Exsanguination, Airway, Breathing, Circulation, Disability, Exposure/Environment.	4/23 (17)

#### Processes and routines in healthcare

Ambulance-specific care pathways, “fast-tracks”, were described in about half (*n* = 13) of the services documents. These range from brief mentions to detailed protocols. Common pathways include stroke, percutaneous coronary intervention (PCI) alerts, hip fractures, trauma, and sepsis. Additional pathways cover psychiatric referrals, neonatal care, and infectious diseases such as COVID-19.

Going through the medical guidelines are explicitly described in most services documents (*n* = 21), ranging from full-day sessions to integrated lectures. Common topics include psychiatric crises, refusal of transport, Intensive care unit (ICU) transfers, and out-of-hospital death protocols. Team roles and individual tasks in the team of two during emergency assignments, as well as the varying levels of emergency priorities (Level 1–3) were also addressed in the documents.

CPR training, described with the Swedish words for ACLS (Advanced Cardiovascular Life Support), BLS (Basic Life Support), or merely as CPR, were specified in 20 organizations documents. Most descriptions include ACLS for both adults and children, while some extend to neonatal and trauma-related CPR. Training also incorporated airway management, mechanical chest compression system, intraosseous access, and emergency medication protocols. Education in meeting the psychological aspects of resuscitation and family support were described in one organization documents.

### Prerequisites for care and nursing

The different induction documents explicitly describe areas that could be summarized as prerequisites for care and nursing. More explicitly learning to handle the medical equipment, medical devices and medications that the ambulance professionals bring with them in the ambulance. In this category, professional approaches were also covered in some documents.

#### Equipment

Twelve services explicitly include training with the medical equipment in the ambulance during the induction period. The level of detail regarding this varied but commonly included content of the different emergency bags, such as equipment to manage sick or injured children, childbirth, bleeding, airway management equipment, infusion warmers, and trauma kits (e.g., tourniquet, decompression needle).

#### Medical devices

In 21 services documents training in handling medical devices such as ventilator, CPAP (Continuous Positive Airway Pressure device), ECG transmission systems and even video laryngoscopy were covered at varying levels including device maintenance and malfunction reporting.

#### Medications

Eleven services explicitly cover knowledge about medications and oxygen therapy, including administration, delegation protocols, and overdose management.

#### Professional approach

A professional approach refers to the way ambulance professionals conduct themselves in their work. Social interaction, personal attitudes, values, ethics and behaviour was highlighted as part of the induction training in five services. One service also involved communication with patients and overcoming language barriers such as using an app-based tool for translation.

### Safe healthcare environment

The category describes the part of the introductory training that aims at providing sustainable conditions and preparedness for a safe care and work environment for both patients and ambulance professionals. The training focuses on management of various types of dangerous substances, healthcare hygiene, personal safety measures, practical handling of threat and risk situations, ergonomics, and patient transfer techniques.

#### Dangerous substances

Most ambulance services (*n* = 16) report that some form of training in chemical, biological, radiological, and nuclear threats, as well as explosive properties (CBRNE), was included in the introductory training. The CBRNE training included theoretical knowledge of various hazardous substances and procedures for handling CBRNE incidents. Practical training was also described as trying on protective masks and suits, as well as handling antidotes.

#### Healthcare hygiene

Basic hygiene routines in prehospital care situations, handling of protective equipment, and how to prevent healthcare related infections to be spread, such as information about personal care and hygiene and washing work clothes were described in many (*n* = 15) of the induction documents.

#### Personal safety measures

Almost all services (*n* = 19) reported that the introductory training includes elements in providing ambulance professionals with conditions for personal safety in the healthcare environment. To provide preparedness for hazardous care situations and to create a security mindset, theoretical knowledge and skill training in preventive conflict management, principles for handling threat and violence situations, self-protection, release techniques, and the function and handling of assault alarms were conveyed.

Training in fire safety, fire protection, fire alarm handling, evacuation and evacuation plans, as well as handling of fire extinguishers and fire drills was described. For personal safety, checklists were also described for example sobriety checks, and action plans in case of suspected alcohol and drug abuse among colleagues.

#### Ergonomics and transfer techniques

All services induction documents (*n* = 23) describe training in ergonomics and patient transfer techniques such as handling the stretcher. Theory and practical training of available aids for transfer (stretcher, motorized stair chair), spinal motion restriction, immobilization equipment, and other patient evacuation techniques were described, as well as how to repair simple defects in the stretcher system.

### Organizational knowledge

The category describes the central and local operational information provided during the induction as well as the training in relevant digital systems that new employees need to work in ambulance services.

#### Central operational information

Almost all ambulance services (*n* = 20) describe that the introductory training began with comprehensive operational information, including organizational structure, decision-making pathways and core values of the organization as well as a presentation of managers, medical directors, the on-call ambulance chief, and other important roles within the organization. Union representatives were also described to provide information about their activities.

New employees were also introduced to terms and conditions of employment, personal planning and scheduling of work shifts, reporting of absences and similar matters, and were given information about employment benefits, occupational health services and crisis support/peer support.

#### Local workplace information

Study visits with local information at the upcoming workplace/station were described to be conducted during the introductory training in several services (*n* = 15). Meeting the local station manager, reviewing various station routines, accessing current contact paths and phone numbers, and taking a tour of the premises were described.

#### Digital workplace systems

During the introductory training, presentation and log-in details for different digital systems such as websites, intranets, and digital software for scheduling and staffing, competence development, healthcare deviations, and medication management were addressed.

### Pedagogical approaches

The documents also displayed that the organizations employed a range of pedagogical approaches to train new employees during the induction. These included classroom lectures, online material, practical skills training, simulation exercises, authorization by a physician, physical on-site visits, assessment by tests, control by checklists, self-studies and providing the new employees with a mentor.

#### Assessment by tests

Different tests to assess the new employee’s knowledge were described in the documents of eleven organizations. These included both mid-programme and final evaluations, and so-called certification exams involving both practical and written assessments. Another assessment described was a physical assessment that included bicycle endurance tests, hand grip strength, core stability, back endurance, lifting strength, simulated stretcher-carrying in stairs, and ability to swim. The term, final exam, either by knowledge test, or skills assessment were described in three organizations documents.

#### Authorization by a physician

Another commonly used (*n* = 12) pedagogical approach described in introductory documents was to be given the authorization to administer specific medications or perform certain tasks independently. Authorization, i.e., a test of knowledge could be conducted as an interview with a physician, a written test, or a combination of both with the aim of having autonomous medication or task authorization.

#### Classroom lectures

Classroom lectures were mentioned as a pedagogical approach in the introductory documents of sixteen organizations. The lectures primarily covered acute conditions such as various medical and surgical conditions, ECG interpretation, childbirth, airway management, and trauma (kinetics and patient care) for both adult and paediatric patients, as well as caring for elderly patients and suicide prevention strategies. Other lectures addressed topics such as threats and violence, personal protective equipment, abusive behaviour, and communication and leadership.

#### Control by checklists

A commonly (*n* = 18) mentioned pedagogical approach was the use of checklists, i.e., written documents to be signed upon completion of various tasks. The checklists were described with varying levels of detail and content, with checkboxes for completion and date or requiring a signature from the supervisor and/or new employee. The different tasks on the checklist could also be assessed at different levels such as theoretical (see or read about) and practical (performed with support or performed independently) level, or be graded as “None, Some, Good, High, Very High.” In one organization, the checklist was proactively used, meaning that it had to be completed before the new employee had his/her first ambulance shift.

#### Mentor

In eight of the organizations documents the pedagogical approach in terms of a mentor or a “buddy” was described to be assigned to the novice professional. Information about the mentorship was deficient. However, a mentor was described as a more experienced ambulance colleague, or someone who had undergone specific training for that role.

#### Online material

Online lectures, pre-recorded material, or web-based training were also commonly used (*n* = 17) as pedagogical approaches. Online materials were utilized for a wide range of topics, including instructional videos on hygiene procedures and equipment usage, lectures on various medical conditions and treatments and informational videos on how to use training portals and intranet navigation. Web-based training focused on process-related education such as CPR, structured patient management, weather and terrain considerations for vehicle operation, or preparatory materials for practical skills training and so-called micro-exercises (theoretical short case-based exercises).

#### Physical on-site visits

Physical visits as a pedagogical approach were described in about half of the organizations’ documents (*n* = 12). Visits included emergency departments and different hospital units such as maternity wards, labs, radiology departments, cardiac intensive care units, and intensive care units. Physical visits could also involve spending a day at a dispatch centre, visiting the police, fire department, primary care centres, local hospitals, or the sea rescue service.

#### Practical skills training

Practical skills training was described as a pedagogical approach in eighteen of the organizations’ introductory documents. Practical skills training involved training to handle the technology of and within the ambulance, vehicle familiarization and emergency driving skills, handling digital equipment, such as the radio communication system, as well as medical and/or immobilization equipment. Putting on and removing protective clothing and equipment was also trained using practical skills training. New employees were also described to go through a period of field training “ride along” before working independently. Other practical training, however mentioned in only two organizations, included team training and prehospital nursing exercises focusing on communication and conversation skills.

#### Self-studies

Self-studies including personal reflection time were mentioned as a pedagogical approach in six of the organizations’ documents. Independent self-study was either required before the start of the introductory days or designated as the employee’s own responsibility to review or familiarize themselves with protocols and guidelines.

#### Simulation exercises

Simulation exercises were identified as a pedagogical approach in the induction materials of thirteen organizations. This sub-category involved various scenarios practiced through simulations, such as CPR and/or trauma scenarios. Notably, in two organizations documents trauma simulation that focused specifically on elderly patients was described. Scenario-based training that focused specifically on communication with children or people with mental health issues was described in one organization’s document. Clinical training centre simulations were mentioned to exist in two organizations. Simulation exercises were also described in terms of joint training with the fire department, involving practicing extracting patients from vehicles, fire extinguishing exercises, and larger multi-centre exercises such as prehospital incident management training. One organization’s document stated that simulation exercises performed with a driving simulator were used on trial.

## Discussion

The result of the analysis process of this study reveals that the content described in induction programmes in Swedish ambulance service was massive with great variations in content. However, the result indicates that there is consensus within the ambulance services. All the included ambulance services induction programmes covered driver training and vehicle knowledge, information transfer, processes and routines in healthcare, as well as ergonomics and transfer techniques (Table 2). Most induction programmes also included prehospital incident management and familiarization with the medical devices in the ambulance. These are areas that may be described as competencies specifically necessary for operational readiness in the ambulance service.

Sweden currently lacks national regulation specifically governing ambulance healthcare, resulting in regional variation in staff educational backgrounds, ambulance equipment, and local medical guidelines [28]. The findings of the present study further indicate variation in how new professionals are inducted to ambulance practice. However, as this study did not evaluate the effectiveness of the identified induction programmes, no conclusions can be drawn regarding whether these differences lead to variations in professional competence, clinical performance, or patient outcomes.

Nevertheless, the observed variation suggests that ambulance services may rely on differing educational priorities, pedagogical assumptions, and local organisational needs when designing induction programmes. From one perspective, a greater degree of standardisation could potentially contribute to more consistent educational conditions for new professionals and support a shared professional foundation across regions. From another perspective, excessive standardisation may risk limiting local adaptation and responsiveness to contextual differences in patient populations, geography, organisational structures, and operational demands. All induction programmes should not necessarily be identical, but rather variation should be balanced with the need to ensure safe and professionally adequate practice.

The above-mentioned variation in induction is also relevant in relation to the concept of equitable care. Equity in healthcare does not imply that care should be identical in all contexts, but rather that patients should have fair access to safe and appropriate care according to their needs [29]. In this context, variation in induction practices does not automatically indicate inequitable care. However, substantial differences in educational conditions, support structures, and opportunities for competence development may contribute to uneven preparedness among new professionals across regions and even countries. Such variation could potentially influence the consistency with which ambulance care is delivered, although further research is required to examine whether and how induction practices affect clinical competence, patient safety, and equity in care [24].

Previous research exploring the experiences of newly employed ambulance professionals has demonstrated that, although novices tend to perceive themselves as competent in the provision of care, they experience uncertainty regarding competencies specific to the ambulance context, such as radio communication, driving large vehicles, navigation, and managing ambulance equipment [18]. Consequently, in the light of the result of this document analysis the induction programmes within ambulance service appear to reflect a shared understanding of the profession’s specific competency requirements, addressing domains in which new professionals themselves report uncertainty.

The results of this study also reveal that some attention was given to competencies of a more interpersonal nature such as nursing skills, professional conduct, and communication abilities, suggesting an emerging but not fully integrated recognition of their relevance within ambulance practice. Since 2016 a fundamental shift from a disease-focused approach towards care that is centred on the individual i.e., person-centred case has been emphasized [30, 31] and research indicates that patients’ sense of safety in the ambulance service is strongly influenced by communication and the nurses’ professional competence [32]. Similarly, Kauppi et al. [33] reported that patients sometimes experience existentially dehumanizing care, leaving them feeling objectified, which further underscores the critical importance of nurses’ attitudes, values, ethics, and behaviour. Despite the recognized importance of these competencies, the ambulance service seems to face challenges in fully implementing person-centred care [34, 35]. The limited attention given to person-centred care within the induction programmes is noteworthy, particularly as person-centredness constitutes a core aspect of nursing practice. The findings may suggest that competence within ambulance care is still predominantly framed in relation to medical and technical capabilities. As a result, other dimensions of nursing competence, such as relational, ethical, and person-centred aspects of care, may become less visible within the educational content. This could be interpreted as reflecting a comparatively narrow conceptualisation of professional nursing competence in the ambulance context. An underuse of nurses’ broader competencies has also been identified as a contributing factor to intentions to leave the profession and, in some cases, actual turnover [36]. This highlights the necessity of more ambulance services incorporating training on interpersonal and professional competencies in their induction programmes, ensuring that new nurses are supported not only in their medical and operational skills but also in developing their professional and interpersonal capabilities.

Furthermore, areas related to the development of professional roles, such as mentorship, personal attitudes, values, ethics, were sparsely described in the induction programmes, and it can be inferred that support and training aimed at helping novice professionals develop a confident professional role is limited during their induction to the ambulance service. In terms of mentorship as part of the induction, this has been called for in nursing literature, and the benefits are well documented [37]. However, in the context of nursing, creating an environment that supports novice nurses in their transition requires substantial commitment from organizations. This includes providing necessary resources, fostering healthy practice environments, and promoting good leadership. Furthermore, it is worth noting that an environment that supports nurses’ transition has been linked to improved collaborative workplaces, higher retention rates, and better patient outcomes [38].

The results of this mapping revealed variation in the pedagogical approaches employed in the induction of newly employed ambulance professionals. This diversity may reflect differing epistemological perspectives on learning and professional development within ambulance services. However, as the present study did not evaluate the effectiveness of the induction programmes or their impact on professional competence, no conclusions can be drawn regarding whether these variations result in differences in competence outcomes or patient safety.

Nevertheless, pedagogical research suggests that the choice of educational strategies may influence learning conditions and opportunities for competence development. For example, traditional classroom lectures alone have been shown to provide limited educational benefits in terms of sustained clinical competence and patient safety outcomes [39], while the pedagogical value of tools such as checklists remains debated. Furthermore, healthcare professionals involved in clinical teaching and supervision are not necessarily formally trained educators, which may create challenges when designing and implementing educational interventions [40]. Contemporary pedagogical research instead highlights the importance of student-active and self-directed learning (SDL) approaches. SDL has been identified as a potential facilitator of professional development among ambulance professionals, provided that structured educational resources and protected time for learning are available during working hours [41]. Research also suggests that case-based training, or real-life experiences should be combined with feedback in a “safe learning atmosphere” to achieve professional competence development and positive effects on patient satisfaction and patient safety [42] When using scenario-based education a permissive approach is of essence. A permissive approach that encourages learners to make mistakes and try again without fear of shame or punitive consequences has been identified as one of the most effective ways to promote deep learning [43].

Consequently, the observed variation in induction practices should not necessarily be interpreted as evidence of unequal competence outcomes, but rather as an indication that ambulance services may rely on differing educational assumptions and pedagogical priorities when introducing new professionals.

One of the most central parts of working in the ambulance service is the ability for clinical reasoning and professional judgement [44]. This type of knowledge, which Aristotle would label Phronesis, can only be developed in the actual context of clinical ambulance service work [15]. However, the educators and senior professionals must be able to support the new professional in reflective dialogues and rather than solely focusing on instrumental, medical and task-oriented practical skills, directing the learning focus of the induction towards *becoming* an ambulance service professional [45]. Research indicates that new professionals who feel socially integrated and clearly understand their role are more likely to be retained and to perform well [46]. Since induction programmes aim to integrate future colleagues rather than students, it may be advantageous to avoid terms such as “student” or “trainee” in onboarding materials. Instead, referring to new professionals as prospective colleagues may help reduce perceived hierarchy and foster a stronger sense of inclusion within the team. This, in turn, can contribute to the development of psychologically safe and cohesive work groups an established success factor in high-stress professional environments such as emergency services [47].

In summary, healthcare professionals involved in induction need pedagogical knowledge to plan and implement educational interventions that are evidence-based, grounded in established learning principles, and designed to ensure that new colleagues acquire the competencies required for their role.

### Study limitations/discussion of methods

Document analysis as a research method can be used to systematically compile and evaluate data in different types of documents, both as paper prints and in digital, internet-based form [26]. Since there were no previous studies that described similar data, the material in the study can be seen as raw data that was analysed in a process to find new knowledge by systematically selecting, evaluating and synthesizing the content that was able to answer the purpose of the study. It should also be mentioned that this study has mapped out the documents and material describing the aimed process of induction, and thereby may be viewed as describing an ideal, whereas what is done in practice may differ.

Document analysis is a cost-effective and accessible method when the desired data already exists. Data from documents is also considered to be stable and accurate and provides good coverage, which can be seen as an advantage [26]. The documents in this study contained a large amount of text that was formulated based on the training needs of the current ambulance service. This could constitute a potential deficiency; details may be missing as the documents were produced for reasons other than to constitute research data. There may also be a risk of “biased selectivity”; the person who published the documents has selected which documents they themselves consider appropriate. However, the advantages of cost-effectiveness and accessibility can be said to outweigh the disadvantages [26].

One region had no formal written introduction material available at the time of data collection. To ensure representation from all participating ambulance services, an interview was conducted with a representative from that region regarding their induction programme. The interview was transcribed verbatim and subsequently treated as a textual document within the document analysis. Although the material differed from the pre-existing organisational documents obtained from the other regions, the transcript constituted a contemporaneous textual account of the introduction practices and was analysed using the same analytical procedure as the remaining material. From a methodological perspective, this approach may be considered acceptable within document analysis [26], as the focus of the analysis concerned the content and description of induction practice rather than the original format of the source material.

During the analysis process, words, sentences and contexts were identified considered to represent the content requested in the study. A careful consideration and interpretation were made to achieve a credible, reliable and representative result. During the analysis, the research group had recurring meetings and discussions to ensure consensus and avoid misunderstandings. All researchers in the group have extensive experience of clinical work within Swedish ambulance care and experience from teaching within ambulance care at university level. This pre-understanding proved to be necessary to understand the material as it contained many specific contextual expressions and formulations in the text that can be difficult to understand for outsiders. However, it is important to rein in your pre-understanding so that it does not influence in the wrong direction and interpret something that is not actually the case.

There was a degree of variation between the ambulance services regarding the number of documents submitted and the extent to which different elements were described. Some ambulance services presented extensive and wordy documentation while some only sent a few documents. However, Bowen [26] describes that it is not the number of documents that determines but the quality of what is in the documents. Based on the material received, no clear indications were found that any major components of the induction programmes had been omitted from the documentation, although in some cases a more detailed description of specific elements would have been desirable to allow for a richer analysis of pedagogical content. Nevertheless, the level of detail was considered sufficient to identify overall content and information of how induction programmes were structured.

## Conclusions

Taken together, the findings of this study suggest a need for more evidence-based and pedagogically coherent induction programmes within the ambulance service. The study highlights the potential value of strengthening the alignment between educational content, pedagogical strategies, and clearly articulated professional competencies. Such competencies should encompass not only technical and clinical skills, but also interpersonal, ethical, and person-centred dimensions of care.

Furthermore, the findings suggest that induction is not solely a matter of knowledge and skill acquisition but also involves processes of professional socialisation. Creating conditions that foster a sense of belonging and psychological safety during induction may therefore be important for supporting retention and the development of cohesive and effective teams within the high-pressure context of ambulance service.

Finally, as the study was based on documentary analysis, it does not allow conclusions to be drawn regarding the effectiveness of different induction approaches or their impact on competence development or patient outcomes. Further research is therefore needed to explore how different pedagogical designs within induction programmes may influence learning processes, clinical competence, and patient safety in ambulance care.

## Data Availability

The data analyzed during the current study are not publicly available due to ownership restrictions, as the introductory materials on which the data are based are owned by the respective ambulance services and are subject to internal policies that limit public distribution.

## Abbreviations

ACLS: Advanced cardiovascular life support
BLS: Basic life support
CBRNE: Chemical, biological, radiological, nuclear threats, explosive properties
CPAP: Continuous positive airway pressure device
CPR: Cardiopulmonary rescue
EMT: Emergency medical technician
METHANE: Major incident, exact location, type of incident, hazards, access, number of casualties, emergency services present and required
PCI: Percutaneous coronary intervention
RAKEL: Radio communication for effective command
RN: Registered nurse
SBAR: Situation, background, assessment, recommendation
SDL: Self-directed learning
WHO: World health organization
